# Simultaneous detection of eight immunosuppressive chicken viruses using a GeXP analyser-based multiplex PCR assay

**DOI:** 10.1186/s12985-015-0455-5

**Published:** 2015-12-30

**Authors:** Tingting Zeng, Zhixun Xie, Liji Xie, Xianwen Deng, Zhiqin Xie, Sisi Luo, Li Huang, Jiaoling Huang

**Affiliations:** Guangxi Key Laboratory of Animal Vaccines and Diagnostics, Guangxi Veterinary Research Institute, 51 Youai North Road, Nanning, Guangxi 530001 China

**Keywords:** GeXP analyser, Multiplex PCR, Immunosuppressive viruses

## Abstract

**Background:**

Immunosuppressive viruses are frequently found as co-infections in the chicken industry, potentially causing serious economic losses. Because traditional molecular biology methods have limited detection ability, a rapid, high-throughput method for the differential diagnosis of these viruses is needed. The objective of this study is to develop a GenomeLab Gene Expression Profiler Analyser-based multiplex PCR method (GeXP-multiplex PCR) for simultaneous detection of eight immunosuppressive chicken viruses.

**Results:**

Using chimeric primers, eight such viruses, including Marek's disease virus (MDV), three subgroups of avian leucosis virus (ALV-A/B/J), reticuloendotheliosis virus (REV), infectious bursal disease virus (IBDV), chicken infectious anaemia virus (CIAV) and avian reovirus (ARV), were amplified and identified by their respective amplicon sizes. The specificity and sensitivity of the optimised GeXP-multiplex PCR assay were evaluated, and the data demonstrated that this technique could selectively amplify these eight viruses at a sensitivity of 100 copies/20 μl when all eight viruses were present. Among 300 examined clinical specimens, 190 were found to be positive for immunosuppressive viruses according to this novel assay.

**Conclusion:**

The GeXP-multiplex PCR assay is a high-throughput, sensitive and specific method for the detection of eight immunosuppressive viruses and can be used for differential diagnosis and molecular epidemiological surveys.

## Background

Immunosuppression causes major economic losses in poultry farming because immunosuppressed chickens are more susceptible to viral and bacterial pathogens, respond poorly to vaccination, and display lower feed conversion efficiency as well as growth retardation. Immunosuppressive chicken viruses include Marek’s disease virus (MDV), avian leucosis virus (ALV), reticuloendotheliosis virus (REV), infectious bursal disease virus (IBDV), chicken infectious anaemia virus (CIAV) and avian reovirus (ARV), all of which affect immune function in chickens and lead to immunosuppression [1–7]. The typical symptoms elicited by these viruses differ, and some symptoms are not readily observable. Immunosuppression in chickens infected with MDV, ALV or REV occurs much earlier than does tumour development and death [1, 2]. Although early infection with IBDV in chicks less than 3 weeks old may not result in the typical symptoms of IBD, this infection nonetheless causes serious immunosuppression [8]. Chickens older than 3 weeks of age are resistant to anaemia after infection with CIAV yet remain susceptible to immunosuppression [9]. Furthermore, the possibility of co-infection makes it difficult to differentiate among these immunosuppressive viruses [10–13].

The detection and differential diagnosis of immunosuppressive viruses are important for the poultry industry. However, conventional methods, such as virus isolation and serum neutralisation tests, are typically time-consuming and labour-intensive procedures [14]. Molecular methods have been used to rapidly detect immunosuppression in chickens, but they are limited by their ability to detect only a few pathogens per reaction [15–20]. Therefore, a rapid, cost-effective, and high-throughput detection technique is needed for the clinical diagnosis of immunosuppressive viral infection in chickens.

The GenomeLab Gene Expression Profiler genetic analysis system (GeXP) is a new multi-target, high-throughput detection platform that integrates RT-PCR or PCR with a labelled, amplified product in a multiplex RT-PCR/PCR assay followed by fluorescence capillary electrophoresis separation based on the sizes of the amplified products [21]. The GeXP profiler utilises gene-specific primers containing 5′-universal adaptor sequences [21]: the chimeric primers consist of a universal sequence fused to the 5′-end of a gene-specific sequence. The forward primer consists of a universal dye-labelled sequence fused to the 5′-end of a gene-specific sequence, whereas the reverse primer consists of a universal unlabelled sequence fused to the 5′-end of a gene-specific sequence. Products differing by 7–10 bp in size are separated by capillary electrophoresis. This technique has been used to identify various diseases in humans, including 11 genotypes of HPV [22]; 9 serotypes of hand, foot, and mouth disease [23]; cancer [21, 24]; 7 enteric viruses [25]; and H1N1 [26]. The GeXP genetic analysis system has also been successfully utilised to simultaneously detect 9 avian respiratory pathogens in clinical samples, 8 swine reproductive and respiratory pathogens and 11 duck viruses [27–29].

In this study, a GeXP-multiplex PCR assay was developed and optimised to simultaneously detect eight immunosuppressive chicken viruses: MDV, ALV (three subgroups of ALV, ALV-A/B/J), REV, IBDV, CIAV and ARV.

## Results

### Specificity of the GeXP-multiplex PCR assay

**Fig. 1:**
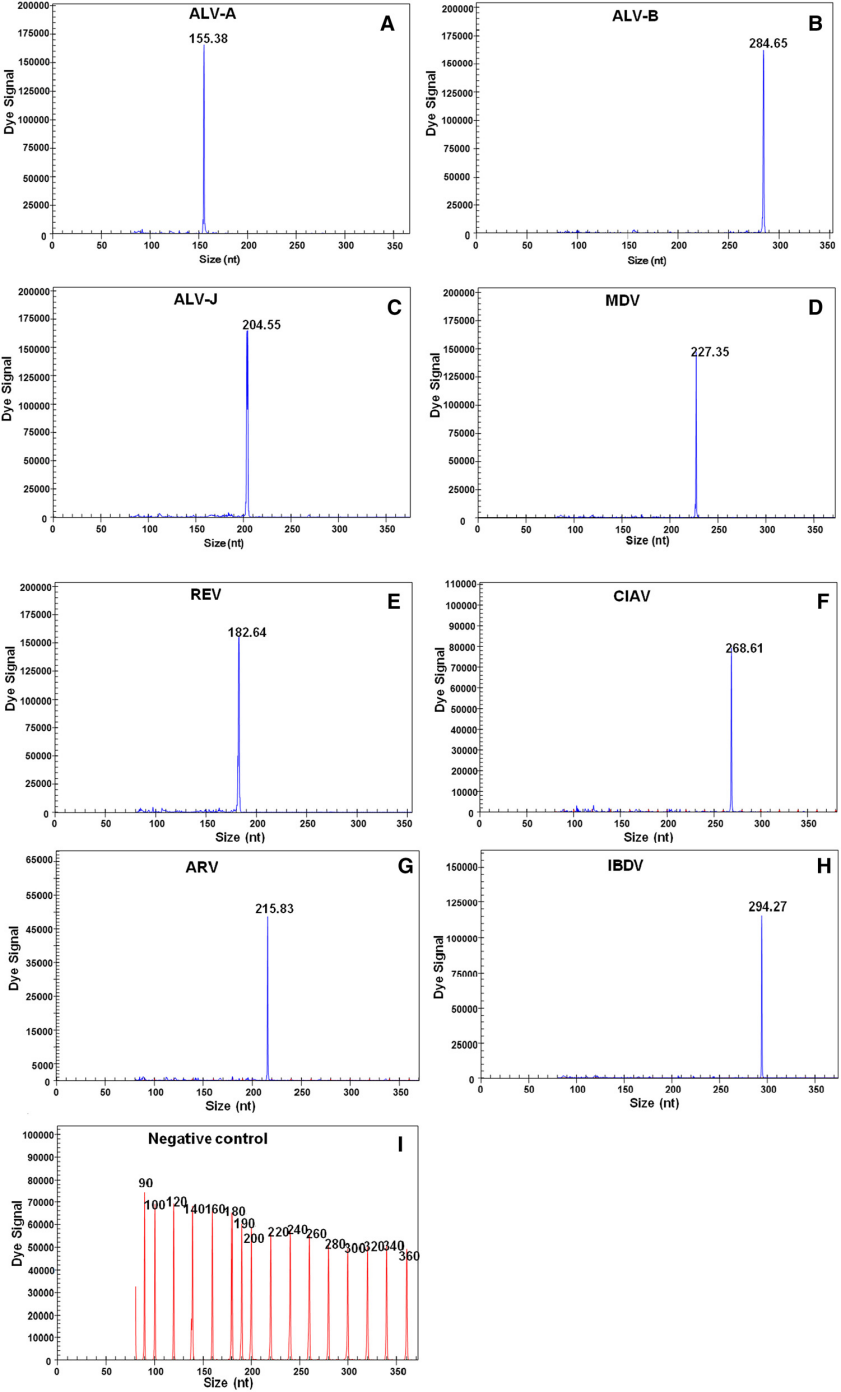
GeXP-multiplex PCR assay specificity. The GeXP-multiplex PCR assay was performed using a single template and mixed primers for the following: ALV-A: 155.38 bp (**a**); ALV-B: 284.65 bp (**b**); ALV-J: 204.55 bp (**c**); MDV: 227.35 bp (**d**); REV: 182.64 bp (**e**); CIAV: 268.61 bp (**f**); ARV: 215.83 bp (**g**); IBDV: 294.27 bp (**h**). DNA from the thymus, spleen and bursa of SPF chickens was used as a negative control (I). The x axes represent the sizes of PCR products in bp, and the y axes represent the dye signal in absorbance units (A.U.). Blue peaks denote specific amplification peaks, and red peaks denote marker peaks

The concentrations of the GeXP-multiplex PCR-specific primers (listed in Table 1) were optimised, and DNA/cDNA from the immunosuppressive viruses described in Table 2 was used individually as a template for evaluating the specificity of the GeXP-multiplex PCR assay. The expected size of each immunosuppressive viral amplicon was determined. A single peak for the complex PCR was detected using the GeXP analyser system (Fig. 1 a-i), and no mispriming with or non-specific amplification of other avian pathogens or the chicken genome was observed.

**Table 1:** Primer sequences and PCR product sizes

Virus	Forward primer sequence (5′–3′)	Reverse primer sequence (5′–3′)	Amplicon size (bp)	Target region	Primer concentration (μM)
MDV	AGGTGACACTATAGAATAAGGGAGCAGACGTACTATGTAGACAA	GTACGACTCACTATAGGGATGGTAAGCAGTCCAAGGGTCA	227	meq	0.16
ALV-A	AGGTGACACTATAGAATACAAGGGGTTCCTTGGTATCT	GTACGACTCACTATAGGGATGTGCCTATCCGCTGTCA	155	gp85	0.2
ALV-B	AGGTGACACTATAGAATATCAATCACGATTCTCCCACC	GTACGACTCACTATAGGGATGTGACGCTTCGTTTACGTCTT	285	gp85	0.2
ALV-J	AGGTGACACTATAGAATACTGATGCAACAACCAGGAAA	GTACGACTCACTATAGGGAGCAGTAACATTAGTGACATACCC	204	gp85	0.2
REV	AGGTGACACTATAGAATAGACCAGGCGAGCAAAATC	GTACGACTCACTATAGGGAGGTGTAATAGGTAGGTATGGAGGA	182	gp90	0.2
IBDV	AGGTGACACTATAGAATAGGGTCAGGGCTAATTGTCTT	GTACGACTCACTATAGGGATCTGTCAGTTCACTCAGGCTTC	294	VP2	0.2
CIAV	AGGTGACACTATAGAATAAAAGGCGAACAACCGATGA	GTACGACTCACTATAGGGATGCCCTGGAGGAAAAGACC	269	VP1	0.2
ARV	AGGTGACACTATAGAATAGGACCCCTACTTCTGTTCTCA	GTACGACTCACTATAGGGAATTTCCCGTGGACGACAT	215	S1	0.16
Universal primers	AGGTGACACTATAGAATA	GTACGACTCACTATAGGGA			0.25

Universal primers sequences are underlined. Chimeric primers were synthesised using universal primers and specific primers

**Table 2:** Sources of pathogens

Pathogen	Source	Pathogen	Source
Reference viruses		Other viruses	
Marek’s disease virus KC453972, KC453973, GX130112, GX140301, 050118, 070123, 090201, 100428	GVRI	Inactivated H5N1 avian influenza virus Re-1	HVRI
Avian leucosis virus subgroup A isolate RSV-1	CVCC	Avian influenza virus H9N6/Duck/HK/147/77	HKU
Avian leucosis virus subgroup A isolate GX110521, GX110522, ALVA01, ALVA02, ALVA03	GVRI	Avian influenza virus H7N2/chicken PA/3979/97	PU
Avian leucosis virus subgroup B isolate RSV-2	CVCC	Newcastle disease virus F48E9	GVRI
Avian leucosis virus subgroup B isolate GX111230, GX130401, ALVB15, ALVB23, ALVB28	GVRI	Newcastle disease virus GX6/02	GVRI
Avian leucosis virus subgroup J isolate KC453974, KC453975, GX090201, GX090521, GX110110, GX120081, GX130018, GX140010	GVRI	Infectious bronchitis virus Massachusetts 41	GVRI
Reticuloendotheliosis virus AV235	CVCC	Infectious laryngotracheitis virus AV1231	CIVDC
Reticuloendotheliosis virus KC453976, KC453977, GX120825, GX131118	GVRI	*Mycoplasma synoviae* CAU0748	CVCC
Avian reovirus S1133, 1733, 526, C78, GuangxiR1, GuangxiR2, GX110058	GVRI		
Infectious bursal disease virus CA, AV162, AV144	CVCC		
Infectious bursal disease virus AV6	CIVDC		
Infectious bursal disease virus 070124, 080113, 090053, 100008, 110110, 130223	GVRI		
Chicken infectious anaemia virus CAU0728, CAU0729, CAU0730, CAU0731, CAU0732	CVCC		
Chicken infectious anaemia virus GXC060821	GVRI		

a) HVRI = Harbin Veterinary Research Institute, China

b) HKU = The University of Hong Kong, China

c) GVRI = Guangxi Veterinary Research Institute, China

d) CIVDC = China Institute of Veterinary Drug Control, China

e) PU = University of Pennsylvania

f) CVCC = China Veterinary Culture Collection Centre

### Sensitivity of the GeXP-PCR assay

**Fig. 2:**
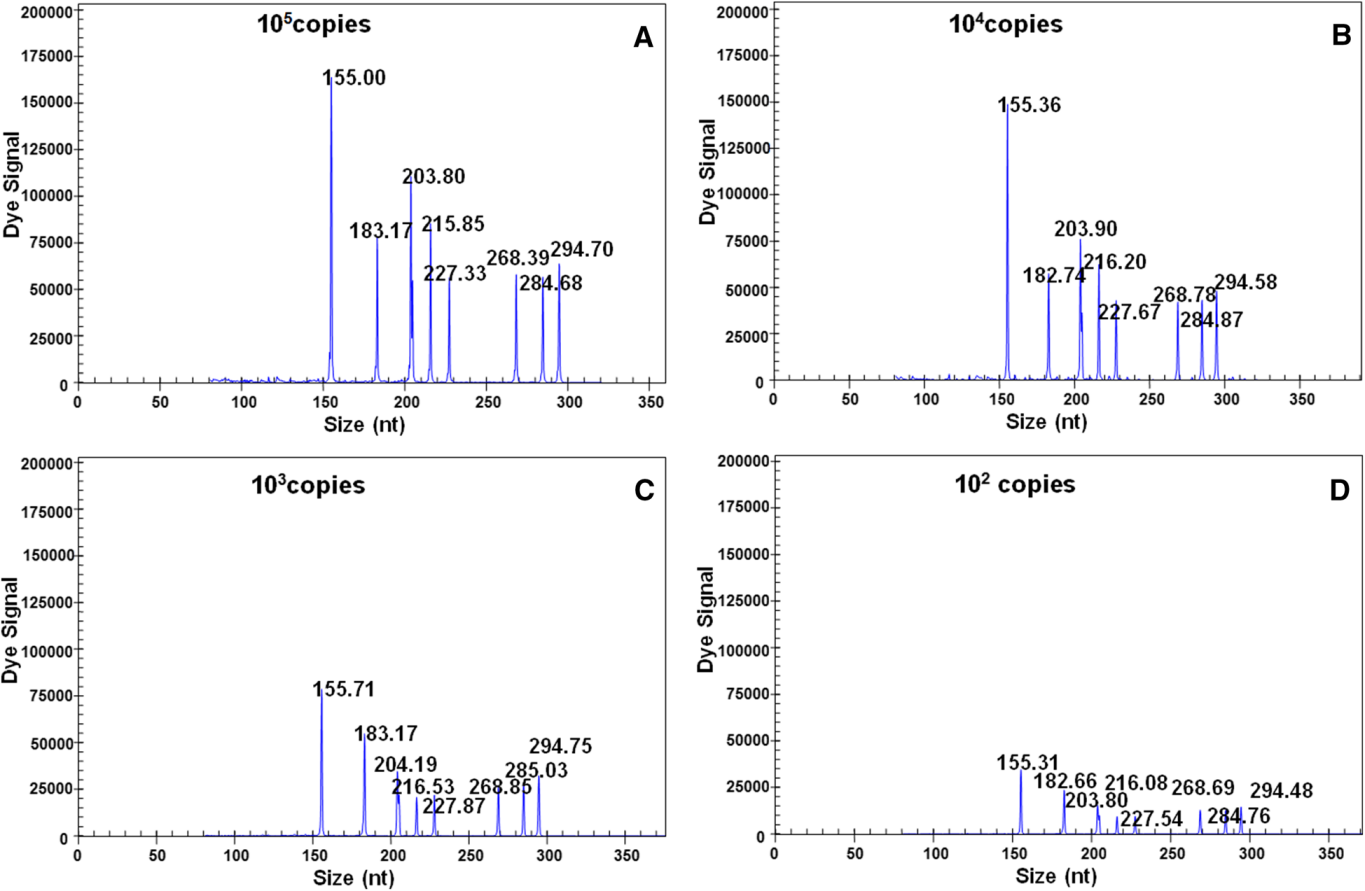
GeXP-multiplex PCR assay sensitivity. The GeXP-multiplex PCR assay was performed with mixed primers using equal amounts of specific DNA-containing plasmid template and *in vitro-*transcribed ssRNA corresponding to eight immunosuppressive viruses at concentrations of 10^5^ (**a**), 10^4^ (**b**), 10^3^ (**c**) or 10^2^ (**d**) copies per reaction. The x axes represent the sizes of PCR products in bp, and the y axes represent the dye signal in absorbance units (A.U.)

The GeXP-multiplex PCR assay achieved the following minimum sensitivity levels in the detection of each of the eight detectable immunosuppressive viruses using eight sets of primers and either plasmid or in vitro ssRNA transcripts: 10 copies for the ALV-A, ALV-J, REV, CIAV and ARV viruses; and 100 copies for the MDV, ALV-B and IBDV viruses (electropherograms not shown). Tenfold serial dilutions of specific DNA-containing plasmids and in vitro ssRNA transcripts from the eight immunosuppressive viruses, i.e., 10^5^ (Fig. 2a), 10^4^ (Fig. 2b), 10^3^ (Fig. 2c) and 10^2^ (Fig. 2d) copies per reaction, were prepared and amplified using an equal amount of template. When all of the pre-mixed, specific DNA-containing plasmids and *in vitro* ssRNA transcripts corresponding to the eight immunosuppressive viruses were present, the GeXP-multiplex PCR assay achieved a minimum sensitivity of 100 copies.

### Artificial mixtures and interference assays

**Fig. 3:**
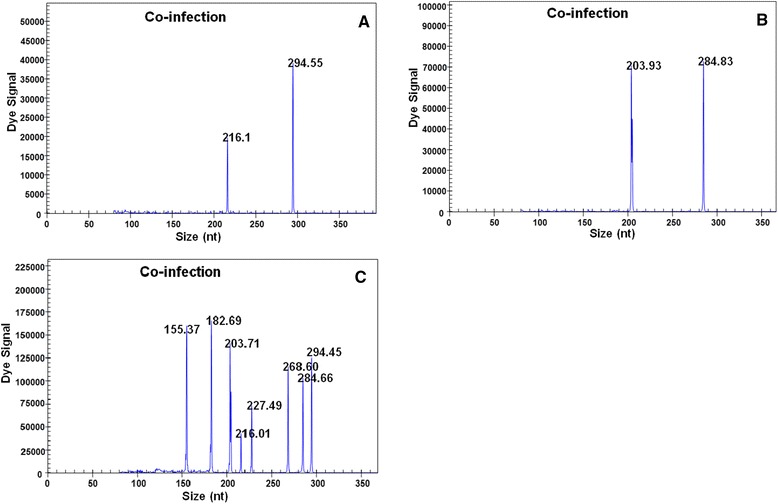
Artificially mixed templates for the GeXP-multiplex PCR assay. The GeXP-multiplex PCR assay was performed using artificially mixed templates and mixed primers for ARV and IBDV (**a**), ALV-J and ALV-B (**b**), or eight immunosuppressive viruses (**c**). The x axes represent the sizes of PCR products in bp, and the y axes represent the dye signal in absorbance units (A.U.)

To test the differentiation ability of the GeXP-multiplex PCR assay, samples previously deemed positive for avian immunosuppressive viruses were randomly mixed; DNA/RNA was then extracted, and the appropriate specific amplification peaks were observed (Fig. 3). When either ARV and IBDV or ALV-J and ALV-B were mixed, two specific amplification peaks were observed (ARV, 216.16 bp; IBDV, 294.55 bp; ALV-J, 203.93 bp; and ALV-B, 284.83 bp), and when DNA/cDNA from all eight immunosuppressive viruses was mixed, eight specific amplification peaks were observed (ALV-A, 155.37 bp; REV, 182.69 bp; ALV-J, 203.71 bp; ARV, 216.01 bp; MDV, 227.49 bp; CIAV, 268.60 bp; ALV-B, 284.65 bp; and IBDV, 294.55 bp).

**Fig. 4:**
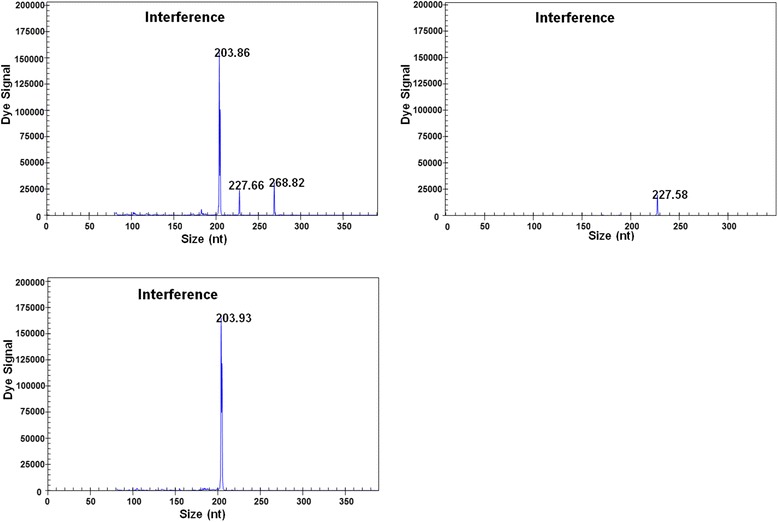
GeXP-multiplex PCR interference assays. The GeXP-multiplex PCR assay was performed using templates for ALV-J, MDV and CIAV (**a**), MDV (**b**), or ALV-J (**c**). The x axes represent the sizes of PCR products in bp, and the y axes represent the dye signal in absorbance units (A.U.)

Specific amplification peaks were also observed when 10^7^ copies of ALV-J, 10^2^ copies of MDV and 10^3^ copies of CIAV were mixed in a single reaction, and similar amplification peaks were observed when 10^7^ copies of ALV-J or 10^2^ copies of MDV were individually tested (Fig. 4). These results demonstrate minimal to no interference by mixed infections in the GeXP-multiplex PCR assay.

### Evaluation of the GeXP-PCR assay using clinical specimens

A total of 300 clinical specimens were collected from diseased farm chickens and tested using the GeXP-multiplex PCR assay. The chickens ranged from 50 to 135 days old, and the cohort included layer, broiler and breeding birds. The results of the GeXP-multiplex PCR assay are provided in Table 3. Independent real-time PCR/RT-PCR and sequencing to identify true positives and negatives confirmed the GeXP-multiplex PCR results, with the positive results being 100 % comparable to those for real-time PCR/RT-PCR and sequencing. Among a total of 190 positive results, 119 specimens displayed a single infection with an immunosuppressive virus. A total of 110 specimens were negative for the eight immunosuppressive viruses. A Kappa value of 1 was found with regard to consistency among GeXP-multiplex PCR, real-time PCR/RT-PCR and sequencing results.

**Table 3:** Detection results for clinical specimens

Single infection						Co-infection					
	Number	Rate (%)	Real-time PCR/RT-PCR results	Sequencing results	Measures of agreement Kappa values		Number	Rate (%)	Real-time PCR/RT-PCR results	Sequencing results	Measures of agreement Kappa values
MDV	26	8.7 %	26	26	1 (*p* < 0.001)	MDV + ALV-A	4	1.3 %	4	4	1 (*p* < 0.001)
ALV-A	6	2.0 %	6	6	1 (*p* < 0.001)	MDV + ALV-J	25	8.3 %	25	25	1 (*p* < 0.001)
ALV-B	2	0.7 %	2	2	1 (*p* < 0.001)	ALV-B + ALV-J	1	0.4 %	1	1	1 (*p* < 0.001)
ALV-J	21	7.0 %	21	21	1 (*p* < 0.001)	MDV + ALV-J + REV	6	2 %	6	6	1 (*p* < 0.001)
REV	15	5 %	15	15	1 (*p* < 0.001)	MDV + CIAV	5	1.6 %	5	5	1 (*p* < 0.001)
IBDV	17	5.7 %	17	17	1 (*p* < 0.001)	MDV + REV	6	2 %	6	6	1 (*p* < 0.001)
CIAV	9	3 %	9	9	1 (*p* < 0.001)	ALV-J + REV	3	1.5 %	3	3	1 (*p* < 0.001)
ARV	23	7.7 %	23	23	1 (*p* < 0.001)	IBDV + ALV-J	5	1.6 %	5	5	1 (*p* < 0.001)
						MDV + CIAV + ALV-J	4	1.3 %	4	4	1 (*p* < 0.001)
						REV + ARV	3	1 %	3	3	1 (*p* < 0.001)
						ALV-J + ARV	6	2 %	6	6	1 (*p* < 0.001)
						ALV-J + IBDV	3	1 %	3	3	1 (*p* < 0.001)

## Discussion

MDV, ALV (three subgroups found predominantly in China, ALV-A/B/J), REV, IBDV, CIAV and ARV are the major immunosuppressive viruses causing economic losses to the chicken industry. Furthermore, the possibility of co-infection increases the difficulty of differentially diagnosing individual viral infections [16, 30–33]. However, conventional diagnostic methods are time consuming, and molecular methods are limited by their ability to detect only a few pathogens per reaction.

The advantages of the GeXP-multiplex PCR assay include its specificity and its high-throughput ability to differentiate eight immunosuppressive viruses. These advantages stem from the use of chimeric and universal primers in a 3-step PCR procedure with different annealing temperatures: the first step amplifies gene-specific sequences within specific regions of the chimeric primers; the second step utilises the entire chimeric primer; and the last step uses universal primers for amplification. As non-specific amplification is minimised by using chimeric primers in the second step at a temperature 13 °C higher than the temperature in step one, only specific amplicons are produced. Furthermore, false-positive reactions are minimised via capillary electrophoresis separation to confirm the identity of the bands [21]. Based on electropherograms, amplicon sizes were found to deviate from their theoretical size by approximately +/-1 bp; for example, amplicons of 154–156 bp indicated a positive result for ALV-A. In contrast, conventional multiplex PCR and multiplex real-time PCR are able to identify only two to four pathogens in one reaction [16, 34–36].

Another advantage of this GeXP-multiplex PCR assay is improved detection sensitivity [27]. In this study, the minimum detection limit of the GeXP-multiplex PCR assay was 100 copies (DNA plasmids or *in vitro* ssRNA transcripts) for all mixed templates, and the minimum absorbance unit (A.U.) was 9000; by default, a reaction was considered positive when A.U. >2000. Further evaluation of clinical specimens confirmed that the positive results were 100 % comparable to the results of sequencing.

The third advantage of this GeXP-multiplex PCR assay is the ability to avoid interference due to the use of universal primers in the last step, ensuring equal amplification efficiency of each target gene, regardless of differences in pathogen concentration.

Of the viruses evaluated in this study, MDV and CIAV are DNA viruses, and IBDV, ALV, REV and ARV are RNA viruses. ALV and REV are retroviruses, which insert their proviral DNA into the host genome for viral replication [37]. In these cases, we recommend using a DNA/RNA kit to extract DNA/RNA together, followed by the procedure described above. All of these immunosuppressive viruses affect the immune organs of chickens, including the thymus, spleen and bursa, and these organs should be collected from diseased chickens for diagnosis.

An analysis of 300 specimens using the GeXP-multiplex PCR assay revealed MDV as the most prevalent single infection, followed by ARV. MDV + ALV-J was the most prevalent co-infection, yet different co-infections were also present. Our data demonstrate that MDV and ALV-J are the main pathogens that co-infect with other immunosuppressive viruses and that various co-infections are common in chickens in south China. Chickens infected with one immunosuppressive virus are more susceptible to attack by another immunosuppressive virus, and vaccine contamination might be a cause of common co-infections [38, 39].

As no assay can be 100 % accurate, there are some limitations to this assay. Virus genomes vary; thus, some primers will likely not be suitable for future use, and additional primers may be required for different variations. Furthermore, the GeXP instrument is expensive for clinical applications; therefore, the availability of less expensive machines will be important.

## Conclusion

In conclusion, the GeXP-multiplex PCR assay is a high-throughput, sensitive and specific method for detecting eight immunosuppressive viruses in chickens. Accordingly, this assay is a potentially useful tool for the detection and differentiation of immunosuppressive viruses and for molecular epidemiologic testing, especially in situations in which chicken flocks are not performing well and the underlying cause may be immunosuppressive viruses. Identifying the immunosuppressive viruses responsible for these infections will be helpful for designing better disease-control programmes.

## Methods

### Ethics statement

This study was approved by the Animal Care and Use Committee (IACUC) of the Guangxi Veterinary Research Institute. Specimen collection from diseased chickens and SPF chickens, the use of chicken embryo fibroblasts (CEFs) and the collection of allantoic fluid from SPF chicken embryos were performed in accordance with the IACUC protocol to minimise animal suffering. The CEFs and embryos were then used for virus propagation.

### Viruses and clinical specimens

MDV, ALV (three subgroups of ALV, A/B/J), REV, IBDV, CIAV, ARV and the other viruses used in this study are listed in Table 2. MDV, ALV-A/B/J, and REV were propagated in chicken embryo fibroblasts (CEFs), and IBDV and ARV were propagated in SPF chicken embryos. CIAV was propagated using a homogenate of positive samples of chicken liver and bone marrow to infect SPF 1-day-old broilers, from which the bone marrow was collected at 10 days after infection. Clinical tissue specimens of the thymus, bursa, spleen, bone marrow, blood and liver were collected from diseased chickens at poultry farms.

### RNA/DNA extraction and RNA reverse transcription

MDV DNA from infected CEFs, CIAV DNA from the bone marrow of affected chickens, REV and ALV-A/B/J genomic proviral DNA from infected CEFs, and IBDV and ARV RNA from allantoic fluid were extracted using the E.Z.N.A.® Total DNA/RNA Isolation Kit (OMEGA, Norcross, GA, USA). The extracted DNA/RNA was eluted in DNase- and RNase-free dH_2_O and stored at −80 °C. The RNA was used to generate cDNA via reverse transcription, as described previously [36].

### GeXP-multiplex PCR primer design

Gene-specific primers were designed based on sequence information obtained from GenBank using Primer premier 5.0 software (Premier, Palo Alto, USA) and NCBI Primer-Blast. ALV-A/B/J primers were designed to correspond to a specific region of the envelope gene gp85, MDV primers to a specific region of the gene encoding Marek’s EcoRI-Q protein (meq), REV primers to a specific region of the envelope gene gp90, IBDV primers to a specific region of the capsid protein gene VP2, CIAV primers to a specific region of the capsid protein gene VP1, and ARV primers to a specific region of the S1 gene. The primer sequences, the sizes of the resulting amplicons, and the target regions are listed in Table 2. The chimeric primers consisted of a universal sequence fused to the 5′-end of a gene-specific sequence. The forward universal primer was labelled at the 5′-end with the fluorescent dye Cy5. All chimeric primers and universal primers were synthesised and purified by polyacrylamide gel electrophoresis (Invitrogen, Shanghai, China).

### GeXP-multiplex PCR assay

GeXP-multiplex PCR assays were performed using the Genome Lab GeXP Starter Kit (Beckman Coulter, Brea, USA) in a 20-μl volume containing 4 μl of 5× buffer, 0.25 μM (final concentration) universal forward and reverse primers, 2 μl of MgCl_2_ (25 μM), 1 μl of chimeric primer mixture, 0.35 μl of Thermo-Start DNA polymerase, 1 μl of cDNA/DNA, and nuclease-free H_2_O. The concentration of the chimeric primers was optimised according to the amplification efficiency of the GeXP-multiplex PCR assay.

The GeXP-multiplex PCR assay was performed via a three-step amplification procedure after a 5-min incubation at 95 °C: 10 cycles of 30 s at 94 °C, 30 s at 55 °C, and 30 s at 72 °C; 10 cycles of 30 s at 94 °C, 30 s at 68 °C, and 30 s at 72 °C; 20 cycles of 30 s at 94 °C, 30 s at 50 °C, and 30 s at 72 °C; and 5 min at 72 °C. The reactions were then held at 4 °C in the thermal cycler (Thermo, Milford, USA).

### Separation by capillary electrophoresis and fragment analysis

After amplification, 1 μl of PCR product was added to 38.75 μl of sample loading solution along with 0.25 μl of DNA size standard 400 (Beckman Coulter, Brea, USA). The fluorescently labelled amplicons were separated into distinct peaks on a electropherogram via GeXP high-resolution capillary electrophoresis and then identified by their respective sizes. The dye signal strength of each peak was measured in A.U. of optical fluorescence and was defined as the fluorescence signal minus the background signal. The data were imported into the analysis module of eXpress Profiler software (Beckman Coulter, Brea, USA) as a tab-delimited file for subsequent analyses.

### Specificity and sensitivity of the GeXP-multiplex PCR assay

The assay specificity for each immunosuppressive viral target was individually tested with a mixture of 8 sets of chimeric primers in a multiplex PCR assay after optimisation. Other conventional chicken viruses, including the H5/H7/H9 serotypes of avian influenza virus (AIV), Newcastle disease virus (NDV), infectious bronchitis virus (IBV) and infectious laryngotracheitis virus (ILTV), were used as negative controls. DNA from the thymus, spleen and bursa of SPF chickens was also used as a negative control.

Specific PCR amplicons for each virus were individually cloned into the pGEM-T vector (Promega, Madison, USA), and the plasmids were purified and sequenced. The sequence data were analysed and compared with the corresponding sequence data in GenBank. The IBDV and ARV plasmids were linearised with SpeI (Takara, Dalian, China) and then *in vitro* transcribed into ssRNA using the RiboMAXLarge Scale RNA Production Systems SP6/T7 Kit (Promega, Madison, USA). After DNase I digestion, ssRNA was purified with TRIzol (Invitrogen, Shanghai, China) and chloroform. The concentration of plasmid DNA and transcribed ssRNA was measured at 260 nm using a NanoDrop 2000 (Thermo Fisher Scientific, Waltham, USA), and the copy number was calculated [40]. Plasmid DNA and transcribed ssRNA were diluted to a final concentration ranging from 10^5^ copies/μl to 1 copy/μl and then subjected to the GeXP-multiplex PCR assay with 8 sets of chimeric primers, both individually and in pre-mixed solutions. The sensitivity of the GeXP-multiplex PCR assay was re-evaluated three times on three different days.

### Artificial mixture and interference assays

To simulate co-infection, thymus, spleen, bursa, bone marrow, blood and liver samples from chickens previously diagnosed with the immunosuppressive viruses were randomly chosen and mixed together in various amounts; DNA/RNA was then extracted. cDNA was generated from RNA as described above. GeXP-multiplex PCR assays were performed using the mixed DNA/cDNA.

Different concentrations of specific DNA-containing plasmids and *in vitro* ssRNA transcripts (10^7^ copies/μl to 10^2^ copies/μl) were used as templates for GeXP-multiplex PCR assays to evaluate interference, and the results were compared with those of single-template GeXP-multiplex PCR assays.

### Evaluation of the GeXP-multiplex PCR assay with clinical specimens

A total of 300 clinical specimens, including the thymus gland, spleen, bursa, bone marrow, blood and liver of diseased chickens, were collected from farms. These diseased chickens showed various symptoms, with most exhibiting depression and anepithymia and some showing extreme emaciation. One-third of the diseased chickens were diarrheic, and approximately half showed respiratory symptoms; some chickens displayed both diarrhea and respiratory symptoms. Approximately one-fourth of the diseased chickens exhibited a poor reaction to vaccination or was prone to bacterial infection. Mortality ranged from 2 % to 30 %. Tumours in the liver, heart, spleen or skin were observed in only approximately 20 chickens and not always in those showing extreme emaciation.

DNA/RNA was extracted, and RNA was reverse transcribed as described above. The GeXP-multiplex PCR assay was performed using DNA/cDNA as the template, and the results were confirmed using independent real-time PCR/RT-PCR and sequencing to determine true positives. Independent real-time PCR/RT-PCR was performed using the primers described above but without the universal sequence at the 5′-end. The results of GeXP-multiplex PCR, real-time PCR/RT-PCR and sequencing were analysed by the Kappa statistical method using SPSS software (IBM, New York, USA).
